# Automatic Cancer Cell Taxonomy Using an Ensemble of Deep Neural Networks

**DOI:** 10.3390/cancers14092224

**Published:** 2022-04-29

**Authors:** Se-woon Choe, Ha-Yeong Yoon, Jae-Yeop Jeong, Jinhyung Park, Jin-Woo Jeong

**Affiliations:** 1 Department of Medical IT Convergence Engineering, Kumoh National Institute of Technology, Gumi 39177, Korea; sewoon@kumoh.ac.kr (S.-W.C.); 20150555@kumoh.ac.kr (J.P.); 2 Department of IT Convergence Engineering, Kumoh National Institute of Technology, Gumi 39177, Korea; 3 Department of Data Science, Seoul National University of Science and Technology, Seoul 01811, Korea; hi.yeong@seoultech.ac.kr (H.-Y.Y.); jaey.jeong@seoultech.ac.kr (J.-Y.J.)

**Keywords:** cancer cell taxonomy, deep learning, convolutional neural network, ensemble approach

## Abstract

**Simple Summary:**

As of recently, cancer is considered a major cause of death in developed and developing countries. Therefore, there is an urgent need for improvements in existing diagnostic methods for effective early diagnosis. However, cross-contamination of cancer cell lines results in the development of inappropriate treatments that cannot be administered to patients. To address this issue, we propose an automatic cancer cell taxonomy with high accuracy using optical images of cells obtained through low-scale benchtop optical microscopy. Specifically, we built a deep-learning-based framework to classify cervical, hepatocellular, breast, and lung cancer cells. The experimental results demonstrated that the proposed deep-learning-based approach facilitates the automatic identification of cancer cells. Moreover, our findings provide important insights into the design of convolutional neural networks for various clinical tasks that utilize microscopic images.

**Abstract:**

Microscopic image-based analysis has been intensively performed for pathological studies and diagnosis of diseases. However, mis-authentication of cell lines due to misjudgments by pathologists has been recognized as a serious problem. To address this problem, we propose a deep-learning-based approach for the automatic taxonomy of cancer cell types. A total of 889 bright-field microscopic images of four cancer cell lines were acquired using a benchtop microscope. Individual cells were further segmented and augmented to increase the image dataset. Afterward, deep transfer learning was adopted to accelerate the classification of cancer types. Experiments revealed that the deep-learning-based methods outperformed traditional machine-learning-based methods. Moreover, the Wilcoxon signed-rank test showed that deep ensemble approaches outperformed individual deep-learning-based models (*p* < 0.001) and were in effect to achieve the classification accuracy up to 97.735%. Additional investigation with the Wilcoxon signed-rank test was conducted to consider various network design choices, such as the type of optimizer, type of learning rate scheduler, degree of fine-tuning, and use of data augmentation. Finally, it was found that the using data augmentation and updating all the weights of a network during fine-tuning improve the overall performance of individual convolutional neural network models.

## 1. Introduction

Recently, cancer has begun being considered a major cause of death in developed and developing countries; thus, the American Cancer Association and GLOBOCAN estimate the number of new cancer cases and deaths each year and aggregate the most recent data on population-based cancer incidence [1,2]. According to the report, 1,806,590 new cancer patients and 606,520 cancer patients were expected to die in the United States by 2020. Specifically, breast cancer is the most common and leading cause of death in women around the world, and the number of patients increases with age, but early diagnosis can increase breast cancer survival by up to 80 percent [3]. Therefore, there is an urgent need for improvements in existing diagnostic methods for effective early diagnosis.

Typically, to diagnose cancer, a radiologist identifies suspicious locations through diagnostic equipment such as X-rays, Magnetic Resonance Imaging (MRI), Computed Tomography (CT), etc., and then conducts a biopsy to check for abnormalities under a microscope [4,5,6,7,8]. Biopsy during clinical diagnosis is an efficient and accurate diagnostic method for cancer detection, and plays an important role in breast cancer as well as in other types of cancer [8,9]. In this approach, a pathologist analyzes a tissue sample of a suspected cancer cell metastasis under a microscope for the detection and classification of tumors. While pathologists familiar with clinical tissues can determine two types of lesions, benign and malignant, manual analysis of microscopic images is a very complex, challenging task, and sometimes misjudged [10]. Therefore, extensive research on computer-aided diagnosis has been actively conducted to increase the accuracy of diagnosis [11,12,13,14,15].

For decades, microscopic image analysis methods have been widely used for biological studies and diagnosis of various diseases, including specific cell counts [16], cell location [12,13], cell shape [17], and cell categorization [14,15]. In particular, microscopic images acquired of tissue or cells facilitate the validation of the presence of certain diseases [18], the categorization of tumor types [19], and the interpretation of cell and molecular genetic mechanisms [20]. However, mis-authentication of cell lines due to cross-contamination has been acknowledged as a serious problem over the past 50 years [21,22]. Generally, cross-contamination of cancer cell lines can be caused by incorrect labeling, cross-use of pipette tips, and sharing of cell culture media [23,24].

Due to the changed or contaminated cell lines, researchers perform experiments using inappropriate cells, resulting in the development of treatments that cannot be administered to patients [25]. Therefore, institutions such as the National Institutes of Health and The International Cell Line Authentication Committee have required additional tests to authenticate the type of cells they are trying to use before conducting relevant research [26,27,28]. Various molecular interpretation trials have been used to solve these problems and identify cell lines, and alternative methods have been actively studied [23,29]. The most widely used at present is short tandem repeat (STR) analysis, which reveals the number of repeated DNA traces of particular DNA motifs [30]. Each sample cell is amplified and processed during STR profiling, and the resulting value is determined to be the same as the standardized cell line profile with approximately 80% similarity [30]. However, STR profiling must be implemented by an experienced professional and is not readily available to users due to its relatively high cost and limited use. In addition, because STR profiling is only suitable for distinguishing cell lines from a single species, researchers need specialized knowledge of the biological differences in each cell [31]. However, even with STR analysis, it was confirmed that 15–20% of the currently used cell lines were incorrectly identified [32]. For example, previous work has confirmed that up to 96 cell lines were misidentified when 482 different human tumor cell lines were analyzed using STR profiling, thus finding that STR profiling alone is insufficient [28]. Therefore, we need an alternative approach for cancer cell line classification that can be easily applied by non-experts in the laboratory, and several artificial-intelligence-based taxonomies of cancer cell lines have been introduced [9,16,17,33].

Recently, convolutional neural networks (CNNs) that can independently extract and construct discriminative features from the data have garnered widespread interest from researchers [34,35,36]. However, in order to utilize images of cells obtained through optical microscopy in deep learning, researchers use expensive and customized equipment such as high-scale microscopy [37], high-frequency single-beam acoustic tweezers [38], hyperspectral imaging systems [39,40], and time-stretch quantitative phase imaging systems [15]. Furthermore, considerable time and effort are required to prepare images stained in various colors as training and test data [9,41].

Therefore, in this work, we propose an automatic cancer cell taxonomy using optical images of cells obtained through low-scale benchtop optical microscopy that is typically used in laboratories. For the automatic classification of four cancer cell types, various deep learning models were trained using a transfer learning approach. We also presented a pipeline for ensemble approach based on both individual deep learning models and multiple heterogeneous models. The main contributions of this study are threefold:

1. We proposed a deep-learning-based approach to prevent cross-contamination of several heterogeneous cancer cell lines.
2. The experimental results showed that the proposed deep-learning-based approach identifies with an accuracy over 97%, demonstrating that our method can be a promising alternative approach to STR for the automated cancer cell taxonomy.
3. We presented and discussed the effects of various design choices on the overall performance of CNN architectures for various clinical tasks that utilize microscopic images.

The rest of this paper is as follows. Section 2 describes the details of the proposed approach. Section 3 presents various experimental results, and Section 4 provides discussions on the experimental results. Finally, we summarize and conclude our work in Section 5.

## 2. Method

**Figure 1:**
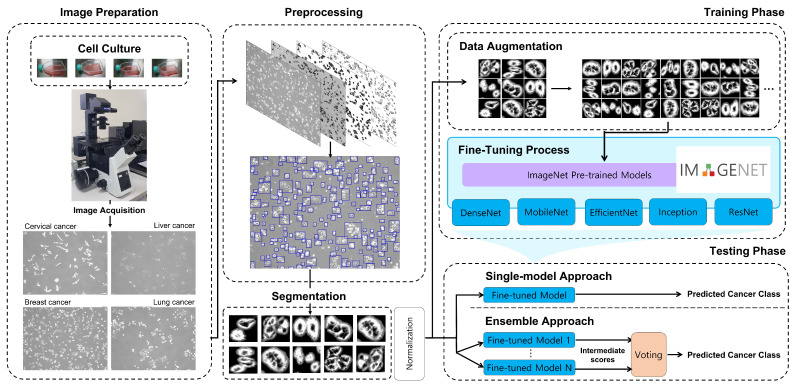
Workflow of the proposed approach.

Figure 1 depicts the proposed framework consisting of four phases: image preparation, image preprocessing, training, and testing of the CNNs. The next subsections describe the details of each step.

### 2.1. Image Preparation

The cancer cell lines were cultured for seven days, and the bright-field images were acquired every day using an inverted fluorescent microscope (IX73 with DP80, Olympus Corp., Tokyo, Japan). Four cells were used in the experiment: HeLa (human, cervical cancer cells), MCF-7 (human, female, 69 years old, Caucasian, breast cancer cells), Huh7 (human, liver cancer cells), and NCI-H1299 (human, lung cancer cells). All cells were purchased from Korean Cell Line Bank (Seoul, Korea) and cultured in the following manner. The cell lines were cultured in a high-glucose Dulbecco’s Modified Eagle Medium containing 10% Fetal Bovine Serum with 1% penicillin streptomycin. The prepared cells were incubated at 37 ${}^{\circ }$C in a humidified incubator with 5% $CO_{2}$ [42]. The prepared cells were trypsinized when 80% confluence was achieved, washed three times with phosphate buffer solution (PBS) [43] to separate the cells, and prepared with an approximate concentration of $1\times 10^{6}$ cells/mL. In total, 889 cell images were collected through the microscope for seven days after starting the cell culture: 247 images in HeLa, 281 images in Huh7, 149 images in MCF7, and 212 images in NCI-H1299.

### 2.2. Image Preprocessing

**Figure 2:**
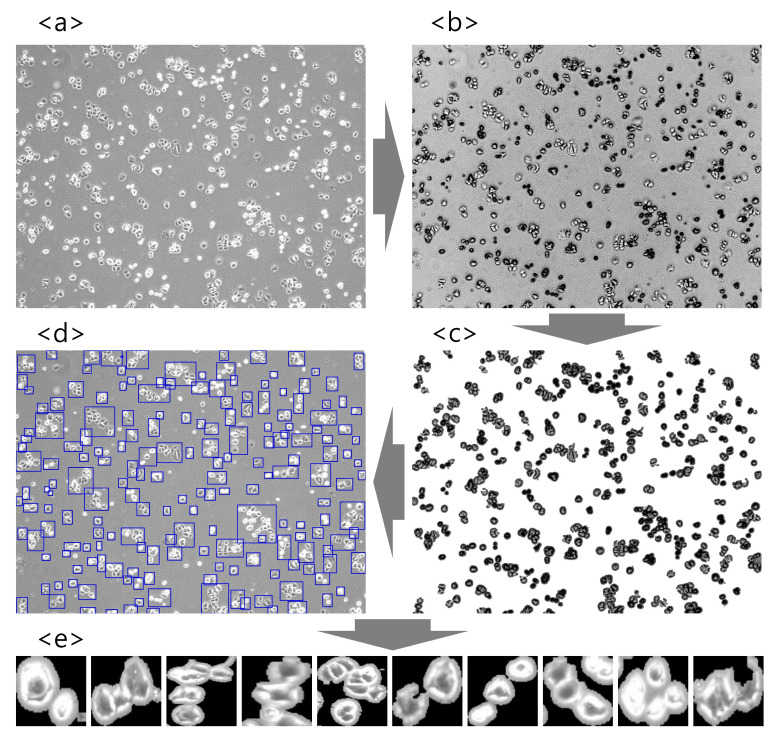
Image preprocessing step. (**a**) Captured microscope image, (**b**) grayscale image, (**c**) noise removed image, (**d**) identified cell contour, (**e**) segmented image patches.

To obtain several morphological types of cell images which will be used for training and testing various CNNs, the acquired images were preprocessed using OpenCV and scikit-image libraries, which are popular open-source libraries used for computer vision and image preprocessing tasks, such as scale transformation, denoising, and adaptive thresholds for Region Of Interest (ROI) of bright field images. The preprocessing steps of the cell images are summarized in Figure 2. First, the brightfield cell image acquired through the microscope (Figure 2a) was converted to grayscale (Figure 2b) and then translated into the binary image using adaptive thresholding. Subsequently, noise removal was performed using the dilation function with a 2 × 2 kernel (Figure 2c). The processed image allows the identification of each cell’s contour and the creation of the bounding boxes (Figure 2d). The size of a bounding box is proportional to the size and number of cells, and uninformative cells or floating debris (the sum of width and height less than 100 pixels) were excluded. The final segmented image patches are depicted in Figure 2e. A total of 27,200 samples were collected by segmenting 889 cell brightfield images. Finally, before feeding the image patches to the CNNs, we apply a different normalization step designed for each CNN architecture that will be introduced in the next subsection. Specifically, the normalization methods include (1) scaling the input pixel values to [0, 1] and then normalizing each channel with respect to the ImageNet, (2) converting the colorspace from RGB to BGR first and then zero-centering the pixel values with respect to the ImageNet without scaling, and (3) scaling the pixel values to [−1, 1] or [0, 1] sample-wise. Therefore, all the images are normalized differently according to the CNN architecture used. More details of the image preprocessing step can be found from Table S1 in Supplementary Data.

### 2.3. Training CNNs for Cancer Classification

Generally, training CNNs from scratch requires a significant amount of data and resources to achieve high performance. Therefore, for efficient training in various domains, transfer learning is widely used, where the weights of a model pretrained on a large-scale dataset are used for solving a new/related task [44]. In this study, we adopted a transfer learning approach wherein the pretrained models are tuned from the general domain (i.e., ImageNet database [45]) to the medical domain (i.e., cancer cell images). Various CNN models pretrained on ImageNet, such as DenseNet121 [46], MobileNetV2 [47], EfficentNetB2 [48], InceptionV3 [49], and ResNet-50 [50], were used as our base networks. All models were trained for 50 epochs using the categorical cross-entropy loss. Moreover, design choices for various learning strategies, such as data augmentation, degree of fine-tuning, optimizer and learning rate scheduler, and ensemble configurations, were considered.

#### 2.3.1. Data Augmentation

**Figure 3:**
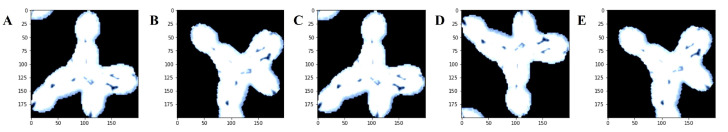
Example of data augmentation: (**A**) original, (**B**) rotation, (**C**) translation, (**D**) vertical flip, (**E**) all.

Data augmentation is considered one of the most promising techniques to improve the robustness and performance of the CNNs by increasing the amount of data with various transformations toward the original images. Of the several available augmentation techniques in the image domain [51], rotation (random rotation between 0–90 degrees), translation (shifting by 2 pixels), and vertical flip methods were considered to adjust spatial parameters of the original image. Figure 3 shows a set of examples, where rotation (Figure 3B), translation (Figure 3C), vertical flip (Figure 3D), and a combination of these methods are applied (Figure 3E) to the original image (Figure 3A), respectively. During the experiments, the original images or images augmented with a combination method (Figure 3E) were used for training CNNs.

#### 2.3.2. Degree of Fine-Tuning

**Figure 4:**
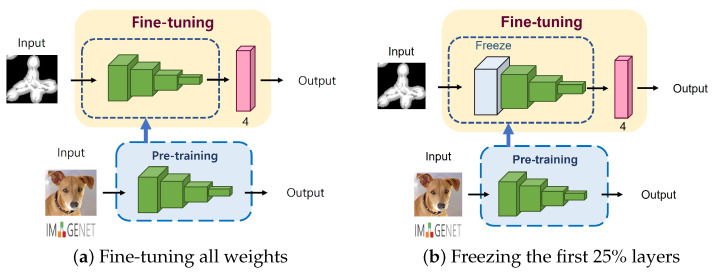
Degree of fine-tuning.

Generally, training deep learning models from scratch requires a large amount of high-quality data as well as computing resources to achieve a high performance. Therefore, transfer learning with fine-tuning has been popular in training deep learning models, as it can transfer the knowledge learned from a large-scale image dataset to the new/similar domain/task. Moreover, it can help to build a more accurate model with less time and data consumed. While adopting a transfer learning method in our pipeline, we considered the following fine-tuning strategies during the training process: (1) updating all parameters in the pretrained model, or (2) freezing the first 25% of the layers in the pretrained model and updating the rest. Figure 4 shows the difference between these strategies. In contrast to the fine-tuning strategy where all the weights are updated to fit a new domain/task (Figure 4a), the second strategy (Figure 4b) utilizes the fixed weight of early layers learned from ImageNet and updates the rest to suit our domain.

#### 2.3.3. Optimizer and Learning Rate Scheduler

An optimizer is one of the most important components that can affect the training speed and accuracy of the CNNs. Of the several available optimizers [52], we selected the Stochastic Gradient Descent (SGD) optimizer, the most popular gradient-descent-based method, and the Adaptive Gradient (AdaGrad) optimzer [53], one of the most popular adaptive methods, as our candidate optimizers. Specifically, the SGD optimizer updates parameters based on the gradient-descent-based optimization using mini-batch data. On the other hand, AdaGrad works similarly to SGD but adaptively controls a learning rate based on the magnitude of previous gradients.

A learning rate is also an important hyper-parameter that determines the extent to which the parameters should be updated. In this study, we used a fixed learning rate of 0.001 or an exponential decay [53] scheduler that allows adaptive scaling of the learning rate at each iteration, which is defined as:

(1) $$\eta \left(t\right)=\eta \left(0\right)\times e^{-t/r}$$

where η(0) is the initial learning rate (0.001), *e* is the decay rate (0.96), *t* is the current step, and *r* is the decay step (10,000). The use of a learning rate scheduler allows an adaptive scaling of the learning rate per each training iteration/epoch.

### 2.4. Ensemble of CNNs

An ensemble approach is a well-known method to improve the performance of a machine-learning-based system by exploiting multiple classification models. A guiding principle in designing ensemble methods has been ’many heads are better than one’ [54]. An ensemble approach typically consists of a set of individual models that predict their own labels for a given sample and therefore can be categorized based on how individual base classifiers are built. Traditionally, in terms of building multiple classifiers, an ensemble approach can be classified into bagging-, boosting-, and stacking-based methods [54]. In bagging, individual base classifiers are trained with a subset of data sampled randomly with replacement [55]. The final prediction is then made by aggregating the result from each base classifier. In this aggregation step, various voting approaches can be considered. Examples of the voting approaches include (1) majority voting (i.e., the predicted target label of the ensemble is the mode of the distribution of individually predicted labels), (2) soft voting (i.e., the predicted target label of the ensemble is the class with the largest sum of probabilities from models), and (3) weighted voting schemes (i.e., the result from each base model is weighted by the model’s importance). Conversely, in the boosting-based method, models are trained sequentially, where subsequent models focus on previous mis-classified samples [56]. Finally, an additional meta-learner can be trained to optimally combine the predictions made by base models in the stacking-based method [57].

From the perspective of a deep learning pipeline, an ensemble approach also can be categorized based on if the ensemble is made across multiple models or within a single model [54]. In the former case, multiple and often independent deep learning models with different model architectures, image preprocessing steps, and pretrained weights are trained and aggregated. Sometimes, each individual model can be trained on a particular subset of training dataset to increase the model diversity. Conversely, the ensemble within a single model is generally achieved by implicit ensembles where a set of neurons, layers, and blocks in the network is deactivated randomly.

**Figure 5:**
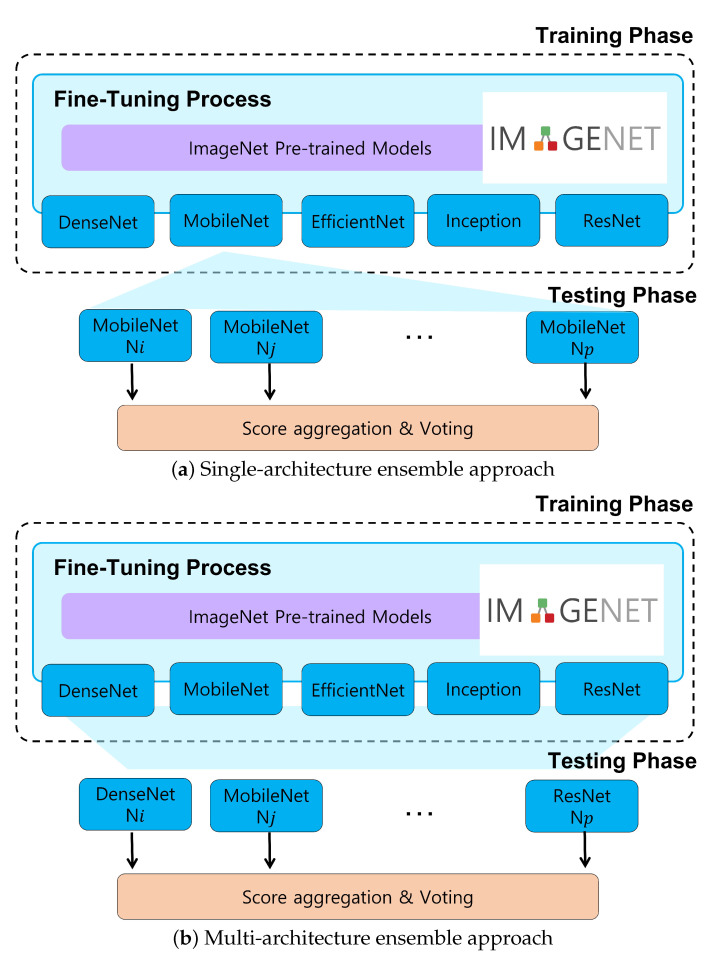
Overview of ensemble approaches.

In this study, we propose two ensemble pipelines for the classification of cancer cells: (i) a single-architecture and (ii) a multi-architecture approach. In the single-architecture approach, a set of the same CNN models trained with different strategies (i.e., different hyper-parameters) is utilized as illustrated in Figure 5a. For example, the MobileNet-based ensemble is composed of a set of MobileNet models trained with different hyper-parameters. Given the test sample, individual MobileNet networks compute their own probabilities for the test sample, and then a voting step is performed to make the final prediction. The multi-architecture ensemble approach is similar to the single-architecture ensemble approach, except that a set of different CNN architectures are included in the ensemble. As depicted in Figure 5b, the class probabilities from different CNN architectures are aggregated for voting. The final prediction is made based on soft voting, where the result is computed by the class probabilities from individual networks. Therefore, our ensemble approach can be considered a kind of bagging ensemble across multiple independent models with soft voting. Additionally, various ensemble configurations are considered to determine the optimal networks to be included in the ensemble. The network selection rule for each ensemble approach is described below:

- Single-architecture ensemble (single-arch, hereafter): As shown in Table 1, there are 16 available configurations for each CNN architecture. In this approach, we select the top-4, top-8, and top-16 best-performing configurations in terms of classification accuracy. Therefore, we can build three ensembles for each model, for a total of 15 single-arch ensemble prediction pipelines.
- Multi-architecture ensemble (multi-arch, hereafter): In contrast to the single-arch pipeline, the multi-arch approach is composed of heterogeneous CNN architectures. To establish this pipeline, we select the top-1, top-2, and top-3 best-performing configurations from each model. Therefore, top-1, top-2, and top-3 multi-arch ensemble pipelines include 5, 10, and 15 individual classification models from different architectures, respectively.

## 3. Experimental Results

### 3.1. Experimental setup

All the experiments were conducted using a GPU server with two NVIDIA RTX 3090 GPUs, 128 GB RAM, and an Intel i9-10940X CPU. We used Tensorflow framework with Keras backend for the training and evaluation of the CNNs. The experiments were conducted using fivefold cross-validation to report precision, recall, accuracy, and F1-score.

### 3.2. Performance evaluation

Table 2 summarizes the performance of the classification models in terms of effectiveness. Note that the reported values are from the best-performing configuration of each CNN model (Table 3). More details of the performance evaluation of all the configurations of each model can be found in Table S2 in the Supplementary Data. In addition to CNNs, we report the performance of traditional machine learning algorithms, such as Support Vector Machine (SVM), Random Forest (RF), Linear Discriminant Analysis (LDA), and K-Nearest Neighbor (k-NN). Similar to the methods proposed in previous studies [58,59], traditional machine learning algorithms used in our experiment were trained with conventional visual features, such as histograms of gradients (HOG), extracted from each cell image separately. The experiments with traditional machine learning algorithms were also conducted using fivefold cross-validation to report precision, recall, accuracy, and F1-score.

**Figure 6:**
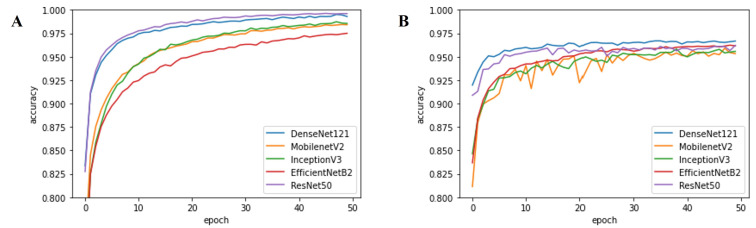
Classification accuracy per epoch: (**A**) training accuracy, (**B**) validation accuracy.

**Figure 7:**
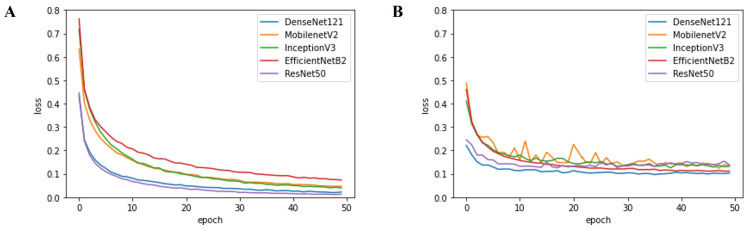
Loss per epoch: (**A**) training loss, (**B**) validation loss.

First, the results in Table 2 clearly demonstrate that traditional machine learning approaches fail to achieve superior performance. Specifically, machine learning methods showed an average accuracy of 49.39%. Conversely, CNNs achieved significant performance gain when compared to the machine learning methods, yielding up to 97.735% classification accuracy (from multi-arch ensemble with the top-3 configuration). Moreover, it is evident that both the single-arch (avg. 96.868%) and multi-arch (avg. 97.657%) ensemble approaches outperformed individual CNN models (avg. 96.071%) in terms of accuracy (*p* < 0.001, Wilcoxon Signed-rank test). Among the ensemble approaches, multi-arch approaches performed better than single-arch approaches, with a performance gain of 0.789%p on average. In the case of individual CNN models, DenseNet121 outperformed the other models with an average performance improvement of 0.844%p in terms of accuracy. Furthermore, the DenseNet121-based single-arch ensemble approach also produced the best result with an accuracy of 97.64%, beating other single-arch models. Finally, Figure 6 and Figure 7 represent the classification accuracy and loss per epoch during training and testing, respectively. As shown in the figures, the validation accuracy and loss of DenseNet121 and ResNet50 converged within 20 epochs, yielding stable performances much earlier than the other networks. The confusion matrices of each individual CNN model with the best-performing configuration are presented in Figure S1 in the Supplementary Data.

Next, we present the number of trainable parameters for each CNN architecture used in the experiments. As shown in Table 4, InceptionV3 and ResNet50 are the heaviest ones with 21–23 M parameters to be updated. In contrast, MobileNetV2 has the smallest number of trainable parameters (∼2.2 M), while DenseNet121 and EfficientNetB2 have 6.7 M and 7.7 M parameters, respectively. Taking into account the number of trainable parameters and classification accuracy, it can be inferred that DenseNet121 would be the best choice for a single CNN model considering that it can provide both moderate model size as well as high effectiveness.

## 4. Discussion

### 4.1. Performance of Deep-Learning-Based Approaches

First, we discuss the effectiveness of each model for automatic cancer cell taxonomy. As summarized in Table 2, all the traditional machine learning approaches failed to achieve superior performance in terms of all the metrics. Specifically, the machine learning methods showed an average accuracy of 49.39%, which is not practical for real-world situations. The SVM classifier yielded the best accuracy of at most 58.7%, which reveals a significant gap between the ML approach and deep learning approaches. Considering that traditional approaches generally utilize a set of classic hand-crafted features, their low performance implies that they are no longer cost-effective. Table 2 also shows that the introduction of deep learning approaches resulted in a significant performance improvement compared to the traditional methods. Moreover, we could observe that the proposed ensemble approach was more effective than individual CNN models for the classification of cancer cells, which was statistically significant (*p* < 0.001). These results imply that CNN models can be effectively applied to the domain of cancer cell microscopic images and can deliver superior performance in the classification of cell types.

On the other hand, Table 2 and Table 4 suggest interesting points regarding the relationship between the classification performance and the model size. It is worth noting that the number of trainable parameters did not significantly affect classification accuracy in the case of our domain. For example, the performance of MobileNetV2 with 2 M parameters and InceptionV3 with 21 M parameters did not show a significant difference (i.e., 95.412% from MobileNetV2 and 95.57% from InceptionV3). Moreover, single-arch ensemble approaches based on MobileNetV2 and InceptionV3 yielded similar classification accuracies of 96.533% and 96.342%, respectively.

### 4.2. Network Design Choice

**Figure 8:**
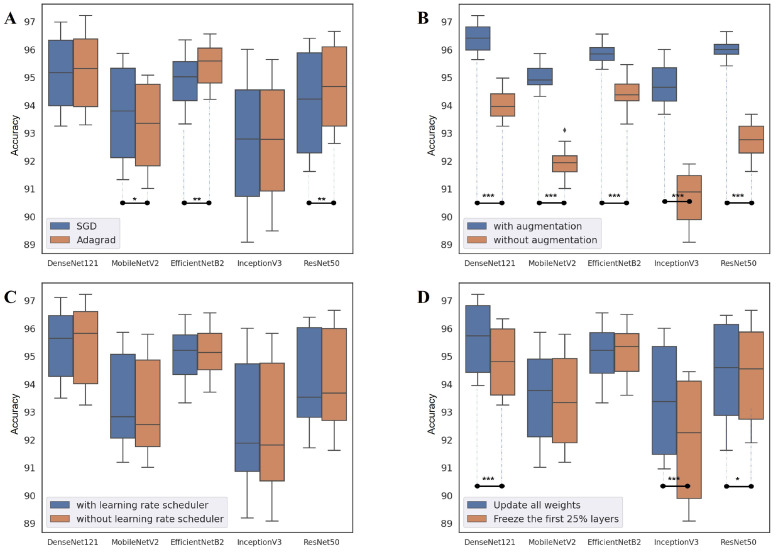
Network design choices (the statistical significance is represented using * (*p* < 0.05), ** (*p* < 0.01), and *** (*p* < 0.001)): (**A**) optimizer, (**B**) data augmentation, (**C**) learning rate scheduler, (**D**) degree of fine-tuning.

In this section, we discuss how the different design choices of each hyper-parameter affect the overall performance in terms of the accuracy of the individual CNN models. The statistical significance based on Wilcoxon Signed-Rank test for each network design choice is depicted in Figure 8 with star marks (* (*p* < 0.05), ** (*p*< 0.01), and *** (*p*< 0.001)).

Optimizer: First, the difference in classification accuracy between the model with the SGD optimizer and the model with the AdaGrad optimizer is presented. As shown in Figure 8A, an optimal choice that worked best for all networks was non-existent. Regardless of the optimizer used, DenseNet121 and InceptionV3 performed equivalently. MobileNetV2 performed better with the SGD optimizer, while EfficientNetB2 and ResNet50 benefited from the use of the AdaGrad optimizer. Therefore, in this domain, an optimizer should be considered based on the type of CNN architecture.

Data augmentation: Second, the effects of the use of data augmentation on the overall performance are presented. In contrast to the use of optimizers, the use of data augmentation significantly affects the overall performance of individual CNN models. As shown in Figure 8B, it is obvious that applying data augmentation improves the classification accuracy of all types of networks (*p* < 0.001). Specifically, the networks with data augmentation achieved an average of 2.85%p higher classification accuracy when compared to those without data augmentation.

Learning rate scheduler: Third, the possible effects of the use of a learning rate scheduler on the performance are discussed. As presented in Figure 8C, it is clear that there is no significant difference in performance between the models with and without the learning rate schedulers. The results indicate that (i) the use of a learning rate scheduler does not significantly affect the performance and (ii) the default choice (0.001) is adequate to achieve high performance.

Fine-tuning: Next, the performance difference between the models trained by updating all weights and the models trained by freezing the first 25% layer and updating just the rest is examined. As shown in Figure 8D, DenseNet121 (*p* < 0.001), InceptionV3 (*p* < 0.001), and ResNet50 (*p* < 0.05) showed significant differences while the performances of MobileNetV2 and EfficientNetB2 were not affected by the degree of fine-tuning.

**Figure 9:**
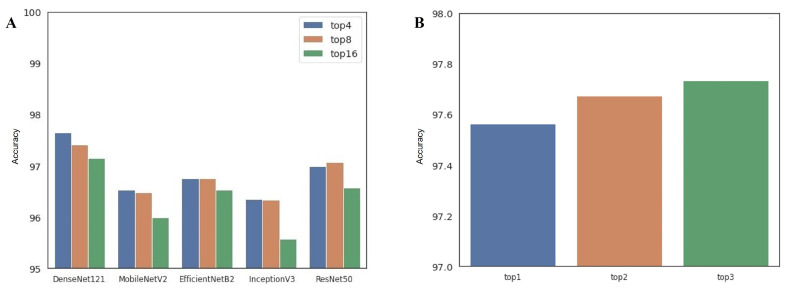
Performance change according to the ensemble configuration: (**A**) single-arch ensemble, (**B**) multi-arch ensemble.

Ensemble: Finally, the possible effects of selection criterion of the ensemble pipeline on the performance of the ensemble prediction are discussed. We first show the difference in performance of the single-arch ensemble approach according to the ensemble configuration. As mentioned in Section 2.4, the single-arch ensemble pipeline can be built using the top-4, top-8, and top-16 models from the same CNN architecture. Figure 9A summarizes the effect of the ensemble configuration on the classification accuracy of the single-arch approach. The result implies that the performance degrades when more networks are involved. Every network showed a similar pattern, where the top-4 or top-8 configuration resulted in the best performance. Basically, the diversity of each base model is important to establish a successful ensemble pipeline. In the case of the single-model approach, the diversity of the base model is relatively low, even though we applied different training strategies, because the base architecture is the same. Therefore, adding more models in this case just resulted in the inclusion of poor models (low-ranked ones), thereby adversely affecting the overall performance. More details on the classification accuracy of each single-arch ensemble approach are presented in Table S3 in the Supplementary Data. In contrast, the multi-arch ensemble pipeline can be built using the top-1, top-2, and top-3 configurations from all types of CNN architectures. Figure 9B shows that the performance of the multi-arch ensemble approach improves as more networks are included in the ensemble. In contrast to the single-arch ensemble approach, the diversity of the models included in the multi-model ensemble is relatively high because their base network architecture and training strategies are totally different. By adding more models in this case, we can include top-performing models with different architectures, thereby increasing the model diversities of the ensemble which can contribute to performance improvement. Finally, Table S4 in the Supplementary Data summarizes a fold-wise classification accuracy of the multi-arch ensemble approach.

### 4.3. Comparison with Previous Studies

Representative CNN studies related to the classification of cancer cells are summarized in Table 5, which shows that our proposed method may provide advantages over the above-mentioned studies. Since the Papanicolaou (Pap) smear test is one of the most essential screening methods for cervical cancer detection [60], it commonly appeared in datasets in the related research [58,61,62,63,64,65]. Despite this popularity, the image acquisition procedure of a Pap smear or a Hematoxylin and Eosin (H&E) stained sample is a labor-intensive and time-consuming process which relies on expert cytologists [58,59,61,62,63,64,65]. In addition, expensive and specialized equipment such as low-coherence off-axis holography [33] or confocal immunofluorescence microscopy [38] is often used to acquire the images, but there is a lack of sufficient datasets to be faced. On the other hand, the strengths of our proposed method include the use of bright field images of the cancer cells from cell culture flasks obtained through the low-scale benchtop optical microscopy that is typically used in laboratories. The other advantage of our method is that it requires no additional wet bench work using fluorescent/staining dyes or biochemical markers. Since the annotated cancer cell lines used in this study were provided directly from cell line provider Korean Cell Line Bank (Seoul, Korea) and cultured in different flasks for each cell line, the training dataset for each cell line serves as the ground truth. Finally, a relatively simple and fast preparation procedure enables researchers to create a large number of datasets for multiple cancer cell lines in their own use. Quantitatively, among the related studies that used the specialized imaging systems, Rubin et al. [33] obtained a maximum accuracy of 90–99% and Oei et al. [38] attained an accuracy of 97.2%. Other studies based on images with staining [58,61,62,63,64,65] reported accuracies of 82.9–96.73%.

Our proposed method achieved a test accuracy of 97.735%, a precision of 97.74, and a recall of 97.74. From these comparisons, it can be inferred that our proposed method outperforms the other classification of cancer cells studies, even though the prepared cancer cell images used in training and evaluation steps require no additional biochemical staining process or expensive image acquisition system compared with these previous studies.

## 5. Conclusions

In this paper, we presented deep-learning-based approaches for the classification of the type of microscopic cancer cell images. We constructed a framework to exploit individual and ensemble CNN pipelines to solve a four-class classification task. The experimental results validated the feasibility of the proposed approach. Specifically, all the CNN models achieved a high classification accuracy of 96.07 (±0.58)%, outperforming traditional machine learning classifiers. In particular, the ensemble approach with a multi-arch strategy achieved the best results, with an accuracy of 97.735%, validating the feasibility of the proposed framework. Moreover, our experimental results indicate that the network design choice and ensemble configuration can affect the overall performance. The results indicated that (i) AdaGrad optimizer is helpful to boost up the performance of EfficientNet-B2 (*p* < 0.01) and RestNet-50 (*p* < 0.01), (ii) data augmentation is always useful for all the networks (*p* < 0.001), (iii) the use of a learning rate scheduler does not make a significant performance difference, and (iv) only DenseNet121 and InceptionV3 benefit from the fine-tuning of all weights rather than freezing part of a network (*p* < 0.001). Based on the experimental results, we believe that the proposed method can reduce the cost of identifying cancer cells, and even users without expertise can identify cell types. Furthermore, our approach does not require expensive equipment and can identify cross-infection among cancer cells using low-scale benchtop microscopy without any additional bench work.

However, additional studies are still required to overcome the limitations of our current approach.

First, four types of cancer cell lines with high mortality were selected to perform label-free cell classification in this study. The annotated cancer cell lines used in this study were provided directly from the official cell line provider, and the same type of cancer cell line was cultured in the individual flask. In other words, pathologically trained experts are not required for validating the test dataset and additional wet bench work to classify cell types. Thus, a relatively simple and fast validation procedure enables us to shorten the preparation time and provide a cost-effective analysis method. On the contrary, randomly mixed cancer cell lines in a single flask may be considered a more realistic model, and it increases the role of the pathologist to validate or identify cell types through fluorescent staining or an H&E staining procedure. Therefore, we plan to apply the proposed framework to mixed-cell images obtained from a single culture flask to provide more practical solutions.

Second, more advanced classification and prediction methods will be required to address various clinical tasks under the aforementioned environments. For example, a transformer architecture, which was very effective for natural language processing tasks, is now widely applied to the computer vision tasks due to its robust and scalable learning capabilities [66,67,68]. In addition, researchers have recently proposed various applications based on self-supervised learning techniques in the computer vision domain and demonstrated effective learning of underlying image representations [69,70,71]. It is also expected that adopting the recent advances in deep learning for computer vision tasks will help in addressing various challenging tasks in the medical domain.

Finally, even though we achieved a higher classification accuracy using ensembles of multiple deep learning architectures with different training strategies, the computational and storage cost required for our models could be another kind of burden for practical use. Therefore, our future work will also focus on improving the computational efficiency as well as classification accuracy by adopting the recent advances in deep learning techniques, for example, knowledge distillation [72,73] from multiple teachers.

## Figures and Tables

**Table 1:** Summary of hyper-parameters.

Parameter	Option	Note
Data augmentation	O	Rotation, translation, and vertical flip
	X	Without any augmentation
Fine-tuning	Without freeze	All weights are updated
	25% freeze	Only 75% of weights are updated
Optimizer	SGD	Stochastic gradient descent
	AdaGrad	Adaptive gradient-based optimization
Learning rate scheduler	O	Exponential decay
	X	Learning rate is fixed to 0.001

**Table 2:** Comparison of the model performance (Best scores in each algorithm are marked in bold).

Algorithm	Model	Accuracy	Precision	Recall	F1-Score
Machine Learning	**SVM**	**58.7** ± 0.74	**58.34** ± 0.76	**58.7** ± 0.74	**58.52** ± 0.75
	RF	49.55 ± 0.32	49.01 ± 0.33	49.55 ± 0.32	49.3 ± 0.32
	LDA	46.26 ± 0.98	44.81 ± 0.98	45.26 ± 0.98	45.03 ± 0.98
	KNN	44.05 ± 0.92	45.86 ± 1.1	44.05 ± 0.92	44.93 ± 0.94
	Average	49.39 ± 5.94	49.51 ± 5.52	49.39 ± 5.94	49.44 ± 5.72
Deep Learning	**DenseNet121**	**96.915** ± 0.072	**96.916** ± 0.077	**96.915** ± 0.072	**96.915** ± 0.075
	EfficientNetB2	96.195 ± 0.23	96.23 ± 0.272	96.176 ± 0.194	96.203 ± 0.232
	ResNet50	96.265 ± 0.138	96.274 ± 0.13	96.265 ± 0.138	96.269 ± 0.134
	InceptionV3	95.57 ± 0.322	95.604 ± 0.376	95.556 ± 0.298	95.58 ± 0.336
	MobileNetV2	95.412 ± 0.223	95.446 ± 0.229	95.412 ± 0.224	95.429 ± 0.226
	Average	96.071 ± 0.584	96.1 ± 0.58	96.06 ± 0.581	96.08 ± 0.58
Ensemble (Single-architecture)	**DenseNet121**	**97.64** ± 0.16	**97.643** ± 0.16	**97.64** ± 0.16	**97.641** ± 0.16
	EfficientNetB2	96.757 ± 0.202	96.763 ± 0.294	96.757 ± 0.294	96.76 ± 0.294
	ResNet50	97.066 ± 0.148	97.073 ± 0.145	97.066 ± 0.148	96.07 ± 0.147
	InceptionV3	96.342 ± 0.196	96.345 ± 0.202	96.342 ± 0.196	96.343 ± 0.199
	MobileNetV2	96.533 ± 0.209	96.55 ± 0.226	96.533 ± 0.209	96.541 ± 0.217
	Average	96.868 ± 0.5	96.875 ± 0.5	96.868 ± 0.5	96.871 ± 0.5
Ensemble (Multi-architecture)	Top-1	97.563 ± 0.145	97.568 ± 0.145	97.563 ± 0.145	97.565 ± 0.145
	Top-2	97.673 ± 0.122	97.677 ± 0.124	97.673 ± 0.122	97.675 ± 0.123
	**Top-3**	**97.735 ± 0.132**	**97.74 ± 0.14**	**97.74 ± 0.132**	**97.74 ± 0.134**
	Average	97.657 ± 0.144	97.661 ± 0.149	97.657 ± 0.144	97.659 ± 0.144

**Table 3:** Configuration of the best-performing individual deep learning models.

Algorithm	Data Augmentation	Degree of Fine-Tuning	Optimizer	Learning Rate Scheduler
DenseNet121	O	All weights	SGD	X
EfficientNetB2	O	All weights	AdaGrad	X
ResNet50	O	All weights	AdaGrad	X
InceptionV3	O	All weights	SGD	O
MobileNetV2	O	Freeze the early 25% layers	SGD	O

**Table 4:** Number of trainable parameters.

	Degree of Fine-Tuning	All Weights	Freeze the First 25% Layers
Model			
DenseNet121		6,957,956	6,716,740
MobileNetV2		2,228,996	2,197,060
EfficientNetB2		7,706,630	7,700,858
InceptionV3		21,776,548	21,348,836
ResNet50		23,542,788	23,315,972

**Table 5:** Comparison with previous studies (“CNN” denotes “Convolutional Neural Network”, “GAN” denotes “Generative Adversarial Network”, “ML” denotes “Machine Learning”, “ANN” denotes “Artificial Neural Network”, “GA” denotes “Generic Algorithm”).

Ref.	Task	Image Acquisition	Method	Num. of Classes	Metric	Performance	Feature
Rubin et al. [33]	Cancer cell classification	Low-coherence off-axis holography without statining	GAN-based approach	4 classes (healthy skin, melanoma cells, colorectal adenocarcinoma colon cells, metastatic colorectal adenocarcinoma cells)	Accuracy	90–99%	CNN feature
Oei et al. [38]	Breast cancer cell detection	Confocal immunofluorescence microscopy images with staining	CNN	2 classes (breast normal cells and cancer cells)	Accuracy	97.2%	CNN feature
Kumar et al. [59]	Cervical cancer cell detection	Microscopic biopsy images with staining	RF, SVM, KNN, fuzzy KNN	2 classese (noncancerous, cancerous)	Accuracy	92.19%	Texture features, morphology and shape features, HOG, wavelet features, etc.
Shi et al. [61]	Cervical cancer cell classification	Microscopic images of Pap smear slides with staining	Graph neural network	5 types of cervical cancer cells (superficial–intermediate, parabasal, koilocytotic, dyskeratotic, and metaplastic cells)	Accuracy	94.93%	CNN feature
Sophea et al. [58]			HOG + SVM	2 classes (normal and abnormal)	Accuracy	94.7%	HOG
Chankong et al. [62]			Bayes, LDA, KNN, ANN, SVM	7 classes (superficial squamous, intermediate squamous, columnar, mild dysplasia, moderate dysplasia, severe dysplasia, and carcinoma in situ)	Accuracy	93.78%	Hand-crafted features (area of cucleus, nucleus-to-cytoplasm ratio, etc.)
Sharma et al. [63]			KNN		Accuracy	82.9%	
Gençtav et al. [64]			Bayesian, decision tree, SVM		Precision	91.7%	
Marinakis et al. [65]			GA		Accuracy	96.73%	
Our proposed method	Cancer cell classification	Microscopic images of cell culture flask without staining	CNN ensemble	4 classes of cell culture flask (HeLa, MCF-7, Huh7, and NCI-H1299)	Accuracy	97.735%	CNN feature

## Data Availability

The data presented in this study are available on request from the corresponding author.

## Supplementary Materials

The following supporting information can be downloaded at: https://www.mdpi.com/article/10.3390/cancers14092224/s1, Table S1: Pseudo code of the image preprocessing step, Table S2: Fivefold cross-validation accuracy of each CNN model with each network configuration, Table S3: Classification accuracy of each single-arch ensemble approach, Table S4: Classification accuracy of the multi-arch ensemble approach, Figure S1: Confusion matrix of each individual CNN model with the best-performing configuration.
