# Supplementary material for: Automatic Cancer Cell Taxonomy Using an Ensemble of Deep Neural Networks
Source: Cancers (Basel). 2022 Apr 29;14(9):2224. doi: 10.3390/cancers14092224 (PMC9100154; doi:10.3390/cancers14092224)

Supplementary Data

Table S1: Pseudo code of the image preprocessing step

```
Algorithm : Image pre-processing
Input: microscopic image
Step 1: load data
        Image ← image; {input image with grayscale}
        cv ← openCV2; {python library for computer vision task}

Step 2: image binarization
        Image ← Image*2; {increase the contrast}
        area ← 7; {block size}
        Image ← cv.threshold(Image, threshold); {image binarization}
        Image ← cv.adaptiveThreshold(Image, area); {local thresholding with area size}

Step 3: noise removal
        kernel ← (2, 2);
        Image ← cv.dilate(Image, kernel); {noise removal with dilation operation using (2, 2) filter}

Step 4: find contours and obtain bounding boxes from the pre-processed image
        contours ← cv.findContours; {each contour consists of four coordinates of cells in the image}
        for each contour in contours do
                bbox ← cv.boundingRect(contour);

Step 5: save image patches
        threshold ← 100;
        for each bbox do
                image_size ← width of bbox + height of bbox;
                if (image_size) > threshold then {small size cell is excluded}
                        save patch;

Output: patches of cell image
```

Table S2: 5-fold cross-validation accuracy of each CNN model with each network configuration. The bold one indicates the winner configuration for each CNN model.

DenseNet121				
Optimizer	Lr scheduler	Augmentation	Degree of fine-tuning	Accuracy (%)
SGD	O	O	All weights	96.798±0.182
**SGD**	**X**	**O**	**All weights**	**96.915±0.072**
SGD	O	O	Freeze the early 25% layers	96.004±0.265
SGD	X	O	Freeze the early 25% layers	95.901±0.083
SGD	O	X	All weights	94.316±0.213
SGD	X	X	All weights	94.371±0.258
SGD	O	X	Freeze the early 25% layers	93.658±0.202
SGD	X	X	Freeze the early 25% layers	93.588±0.243
Adagrad	O	O	All weights	96.761±0.232
Adagrad	X	O	All weights	96.868±0.212
Adagrad	O	O	Freeze the early 25% layers	96.048±0.269
Adagrad	X	O	Freeze the early 25% layers	96.085±0.144
Adagrad	O	X	All weights	94.518±0.264
Adagrad	X	X	All weights	94.342±0.349
Adagrad	O	X	Freeze the early 25% layers	93.717±0.189
Adagrad	X	X	Freeze the early 25% layers	93.577±0.220
MobileNetV2				
Optimizer	Lr scheduler	Augmentation	Degree of fine-tuning	Accuracy (%)
SGD	O	O	All weights	95.272±0.381
SGD	X	O	All weights	95.287±0.617
**SGD**	**O**	**O**	**Freeze the early 25% layers**	**95.412±0.223**
SGD	X	O	Freeze the early 25% layers	95.276±0.408
SGD	O	X	All weights	92.496±0.381
SGD	X	X	All weights	91.982±0.567
SGD	O	X	Freeze the early 25% layers	92.07±0.339
SGD	X	X	Freeze the early 25% layers	91.801±0.148
Adagrad	O	O	All weights	94.849±0.261
Adagrad	X	O	All weights	94.757±0.123
Adagrad	O	O	Freeze the early 25% layers	94.629±0.285
Adagrad	X	O	Freeze the early 25% layers	94.757±0.205
Adagrad	O	X	All weights	91.908±0.28
Adagrad	X	X	All weights	91.588±0.499
Adagrad	O	X	Freeze the early 25% layers	91.732±0.332
Adagrad	X	X	Freeze the early 25% layers	91.897±0.287
EfficientNetB2				
Optimizer	Lr scheduler	Augmentation	Degree of fine-tuning	Accuracy (%)
SGD	O	O	All weights	95.702±0.385
SGD	X	O	All weights	95.64±0.219
SGD	O	O	Freeze the early 25% layers	95.658±0.402
SGD	X	O	Freeze the early 25% layers	95.603±0.210
SGD	O	X	All weights	93.949±0.398
SGD	X	X	All weights	94.162±0.341
SGD	O	X	Freeze the early 25% layers	94.125±0.433
SGD	X	X	Freeze the early 25% layers	94.191±0.331
Adagrad	O	O	All weights	95.96±0.218
**Adagrad**	**X**	**O**	**All weights**	**96.195±0.23**
Adagrad	O	O	Freeze the early 25% layers	96.044±0.301
Adagrad	X	O	Freeze the early 25% layers	96.085±0.269
Adagrad	O	X	All weights	94.64±0.306
Adagrad	X	X	All weights	94.783±0.279
Adagrad	O	X	Freeze the early 25% layers	94.665±0.514
Adagrad	X	X	Freeze the early 25% layers	94.813±0.298
InceptionV3				
Optimizer	Lr scheduler	Augmentation	Degree of fine-tuning	Accuracy (%)
**SGD**	**O**	**O**	**All weights**	**95.57±0.322**
SGD	X	O	All weights	95.515±0.319
SGD	O	O	Freeze the early 25% layers	94.077±0.176
SGD	X	O	Freeze the early 25% layers	94.088±0.288

SGD	O	X	All weights	91.658±0.194
SGD	X	X	All weights	91.382±0.343
SGD	O	X	Freeze the early 25% layers	89.500±0.329
SGD	X	X	Freeze the early 25% layers	89.438±0.236
Adagrad	O	O	All weights	95.206±0.339
Adagrad	X	O	All weights	95.136±0.324
Adagrad	O	O	Freeze the early 25% layers	94.114±0.256
Adagrad	X	O	Freeze the early 25% layers	94.158±0.327
Adagrad	O	X	All weights	91.382±0.353
Adagrad	X	X	All weights	91.349±0.402
Adagrad	O	X	Freeze the early 25% layers	90.324±0.306
Adagrad	X	X	Freeze the early 25% layers	90.151±0.375
ResNet50				
Optimizer	Lr scheduler	Augmentation	Degree of fine-tuning	Accuracy (%)
SGD	O	O	All weights	96.114±0.177
SGD	X	O	All weights	95.919±0.276
SGD	O	O	Freeze the early 25% layers	95.794±0.194
SGD	X	O	Freeze the early 25% layers	95.662±0.219
SGD	O	X	All weights	92.360±0.388
SGD	X	X	All weights	92.118±0.448
SGD	O	X	Freeze the early 25% layers	92.382±0.441
SGD	X	X	Freeze the early 25% layers	92.195±0.1
Adagrad	O	O	All weights	96.114±0.192
**Adagrad**	**X**	**O**	**All weights**	**96.265±0.138**
Adagrad	O	O	Freeze the early 25% layers	95.996±0.283
Adagrad	X	O	Freeze the early 25% layers	96.066±0.338
Adagrad	O	X	All weights	93.324±0.184
Adagrad	X	X	All weights	93.228±0.302
Adagrad	O	X	Freeze the early 25% layers	93.147±0.316
Adagrad	X	X	Freeze the early 25% layers	93.305±0.355

Table S3: Classification accuracy of each single-model ensemble approach

DenseNet121			MobileNetV2			EfficientNetB2		
Fold	Ensemble	Accuracy (%)	Fold	Ensemble	Accuracy (%)	Fold	Ensemble	Accuracy (%)
1	Top 4	97.592	**1**	**Top 4**	**96.71**	1	Top 4	96.857
1	Top 8	97.371	1	Top 8	96.691	1	Top 8	96.857
1	Top 16	96.893	1	Top 16	96.213	1	Top 16	96.6544118
**2**	**Top 4**	**97.813**	2	Top 4	96.691	2	Top 4	97.040
2	Top 8	97.426	2	Top 8	96.581	**2**	**Top 8**	**97.188**
2	Top 16	97.371	2	Top 16	96.158	2	Top 16	96.985
3	Top 4	97.757	3	Top 4	96.618	3	Top 4	96.526
3	Top 8	97.371	3	Top 8	96.507	3	Top 8	96.507
3	Top 16	97.298	3	Top 16	96.342	3	Top 16	96.397
4	Top 4	97.629	4	Top 4	96.213	4	Top 4	96.618
4	Top 8	97.5	4	Top 8	96.25	4	Top 8	96.489
4	Top 16	97.114	4	Top 16	95.643	4	Top 16	96.232
5	Top 4	97.408	5	Top 4	96.434	5	Top 4	96.746
5	Top 8	97.39	5	Top 8	96.342	5	Top 8	96.728
5	Top 16	97.077	5	Top 16	95.588	5	Top 16	96.397

InceptionV3			ResNet50		
Fold	Ensemble	Accuracy (%)	Fold	Ensemble	Accuracy (%)
1	Top 4	96.195	1	Top 4	96.949
1	Top 8	96.452	1	Top 8	97.022
1	Top 16	95.680	1	Top 16	96.360
**2**	**Top 4**	**96.563**	2	Top 4	97.077
2	Top 8	96.526	**2**	**Top 8**	**97.261**
2	Top 16	95.919	2	Top 16	96.765
3	Top 4	96.415	3	Top 4	97.059
3	Top 8	96.268	3	Top 8	96.930
3	Top 16	95.441	3	Top 16	96.746
4	Top 4	96.085	4	Top 4	96.967
4	Top 8	96.195	4	Top 8	97.022
4	Top 16	95.239	4	Top 16	96.434
5	Top 4	96.452	5	Top 4	96.875
5	Top 8	96.195	5	Top 8	97.096
5	Top 16	95.588	5	Top 16	96.563

Ensemble	Model				
	DenseNet121	MobileNetV2	EfficientNetB2	InceptionV2	ResNet50
Top 4	**97.64±0.16**	**96.533±0.209**	**96.757±0.202**	**96.342±0.196**	96.985±0.083
Top 8	97.412±0.054	96.474±0.178	96.754±0.287	96.327±0.153	**97.066±0.13**
Top16	97.151±0.19	95.989±0.348	96.533±0.295	95.574±0.255	96.574±0.181

Table S4: Classification accuracy of the multi-model ensemble approach

Cross ensemble		
Fold	Ensemble	Accuracy (%)
1	Top 1	97.482
1	Top 2	97.665
1	Top 3	97.665
2	Top 1	97.776
2	Top 2	97.794
**2**	**Top 3**	**97.904**
3	Top 1	97.647
3	Top 2	97.794
3	Top 3	97.831
4	Top 1	97.482
4	Top 2	97.518
4	Top 3	97.574
5	Top 1	97.426
5	Top 2	97.592
5	Top 3	97.702

Figure S1: Confusion matrix of each individual CNN model with the best-performing configuration

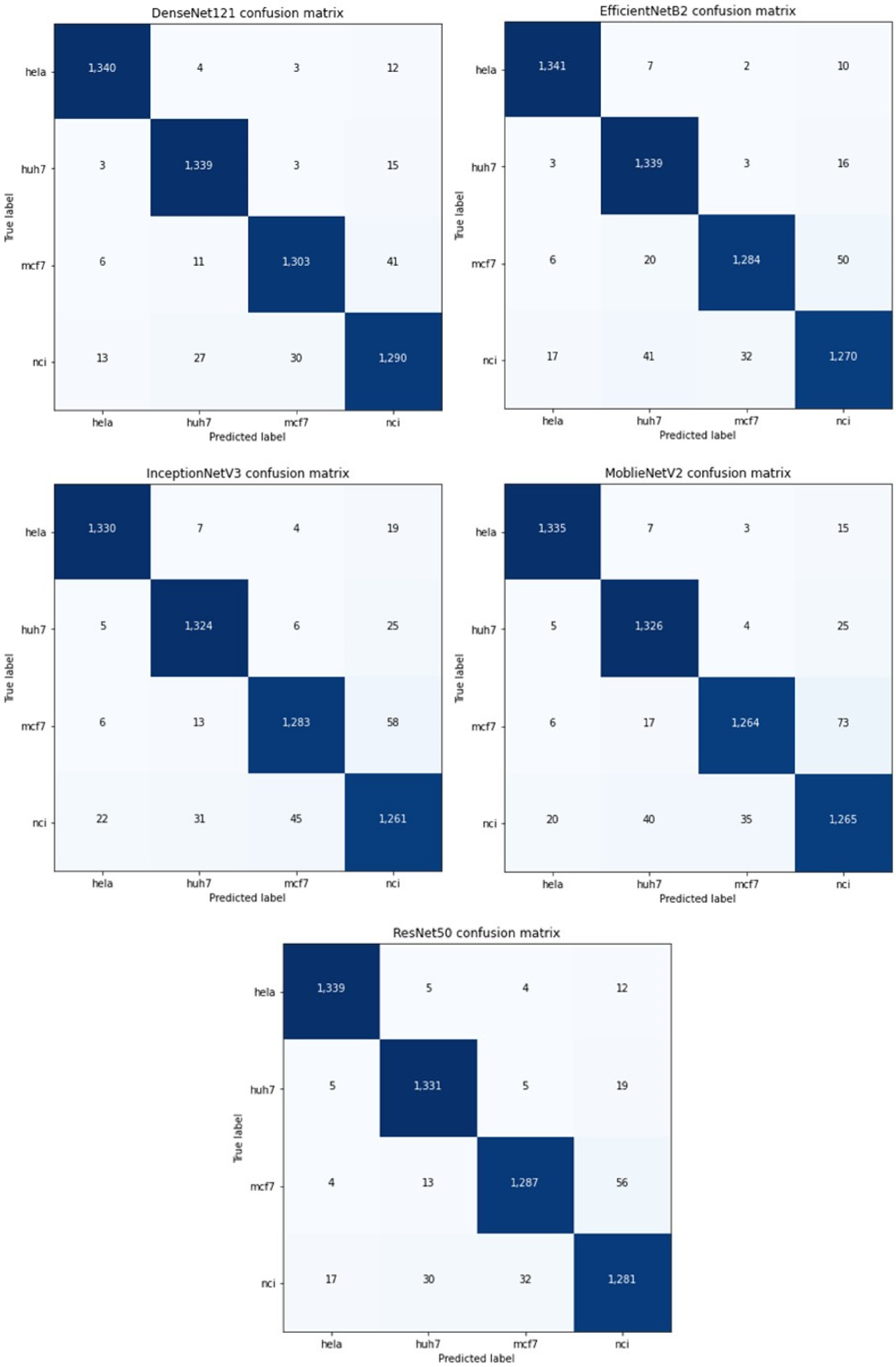

Supplement: Supplementary file 1 [file cancers-14-02224-s001.zip › cancers-1459165-supplementary.pdf]
